# Validating the German Version of the Quality of Relationship Inventory: Confirming the Three-Factor Structure and Report of Psychometric Properties

**DOI:** 10.1371/journal.pone.0037380

**Published:** 2012-05-25

**Authors:** Iris Reiner, Manfred Beutel, Christian Skaletz, Elmar Brähler, Yve Stöbel-Richter

**Affiliations:** 1 Department of Psychosomatic Medicine and Psychotherapy, Johannes Gutenberg-University Mainz, Mainz, Germany; 2 Department of Mental Health, University Hospital Leipzig, Leipzig, Germany; Catholic University of Sacred Heart of Rome, Italy

## Abstract

Research on psychosocial influences such as relationship characteristics has received increased attention in the clinical as well as social-psychological field. Several studies demonstrated that the quality of relationships, in particular with respect to the perceived support within intimate relationships, profoundly affects individuals' mental and physical health. There is, however, a limited choice of valid and internationally known assessments of relationship quality in Germany. We report the validation of the German version of the Quality of Relationships Inventory (QRI). First, we evaluated its factor structure in a representative German sample of 1.494 participants by means of confirmatory factor analysis. Our findings support the previously proposed three-factor structure. Second, importance and satisfaction with different relationship domains (family/children and relationship/sexuality) were linked with the QRI scales, demonstrating high construct validity. Finally, we report sex and age differences regarding the perceived relationship support, conflict and depth in our German sample. In conclusion, the QRI is a reliable and valid measurement to assess social support in romantic relationships in the German population.

## Introduction

Multidisciplinary research has shown that the quality in relationships impacts psychological and physical health [1], [2]. In particular, support plays a major role in most theories of close relationships [3], [4] and a considerable amount of studies suggests that perceived support is an important determinant of relationship satisfaction as well as psychological and physical well-being [5], [6], [7]. The Quality of Relationships Inventory, QRI [8], is a widely used self-report questionnaire of perceived support. It assesses relationship-specific perceptions of social support, consisting of people's expectations about the availability of support from particular significant others [8], [9]. The QRI focuses on support perceived from a particular source, e.g. within an intimate relationship, rather than reflecting a person's perceived support from any individual in his or her social network. Moreover, the QRI includes an assessment of two other features highly relevant to relationship quality: Conflict (the extent to which the relationship is a source of conflict, angry and ambivalent feelings) and depth (the importance of the relationship). So far, the QRI has proven useful in both clinical and nonclinical research on close relationships: It shows very high correlation with other assessments of subjectively perceived relationship satisfaction as well as behavioral relationship quality (in terms of specific and positive, relationship-enhancing behaviors [10] Although the QRI is mostly used to assess the quality of romantic and intimate relationships, it has been successfully applied in the assessment of the quality of other relationship such as mentoring or peer relationships [11], [12].

The significant meaning of perceived support for relationship quality is a cross-cultural phenomenon [13]. With growing research on the importance of high support and relationship quality on health benefits including lower morbidity [14], [15], [16], [17], [18], valid and reliable known assessments are needed. Moreover, in order to compare study results cross-culturally, the application of internationally used and known instruments are of advantage.

In many studies, the QRI has been applied to assess relationship quality and its meaning for physical and mental health outcomes [19], [20], [21], [22]. The QRI has been mostly used in US samples, but has also been applied in European and Asian countries, e.g. in a comparative international study on the association between irritable bowel syndrome and the quality of relationship [23]. To our knowledge, however, information on QRI validation and psychometric properties is only available from two countries outside the US: In a representative sample of 286 Belgian couples, the three factor structure of the QRI could be confirmed [24]. In a small Japanese cohort study of 40 childless couples who had had two recurrent spontaneous abortions, exploratory factor analysis on the QRI revealed a two-factor structure, representing supportive and conflictual features [25].

To date, there is no validated German version of the QRI. Moreover, psychometric properties of the QRI have been derived from rather small couple, student or clinical samples [8], [24], [26]. Thus, psychometric QRI data from a large representative sample are required. Moreover, little is known about relationship quality as reflected in perceived support, conflict and depth in German individuals who currently live in a romantic partnership. In order to close these research gaps, the aims of the present study were the following:

1. Investigation the factor structure of the QRI by means of confirmatory factor analysis (CFA) in a sample of German adults who are currently in a committed and cohabiting heterosexual relationship.
2. Validation of the QRI scales with external criteria (assessment of importance/significance of differential relationship aspects).
3. Report of psychometric properties (differences regarding age, sex and parenthood) of the QRI scales (Support, Conflict, and Depth) in a large representative German sample.

## Methods

### Sample Recruitment and Procedure of data collection

A representative sample of the German population was recruited in November and December 2009, with assistance by an independent agency specializing in market, opinion, and social research (USUMA, Berlin, Germany) in order to explore various health and social attitudes and behaviors in Germany. A three-stage random sampling procedure was used to select (1) sample point regions from 258 regions that were determined based on representative data; (2) target households within sample point regions using a random route procedure; and (3) target persons within target households according to a kish selection grid. Inclusion criteria were age ≥14 years and fluent German. Following this procedure, 4069 noninstitutionalized civilians were randomly selected from all German states. Of these, N = 2520 individuals participated in the assessment, corresponding to a response rate of 61.9% (398 [9.8%] households could not be reached; 539 [13.3%] refused to participate; 160 [3.9%] target persons could not be reached; 11 [0.3%] target persons were incapacitated; and 441 [10.8%] refused to participate). Detailed information on recruitment and data collection procedure are provided by Hauser et al. [27]. All participants were visited in-person, informed about the study procedures by a trained research assistant, and signed an informed consent prior to assessment (for minor participants, informed consent was additionally obtained from one parent). Participants who were currently in a committed and cohabiting heterosexual relationship (n = 1517) were given the QRI. All of them were of full age (≥18). It is worth mentioning that participants were not related to each other. Thus, unrelated men and women and not couples were surveyed. In our study sample, only participants with no missing items in the QRI were included (n = 1494).

All interviewers/researchers involved were aware of the responsibility for confidentiality in respect to participants' records. The data used were de-identified. The study adhered to the ethical guidelines of the ICC/ESOMAR International Code of Marketing and Social Research Practice. The present study posed a low risk to the participants. An additional ethical approval was not required as procedures including medical treatments, invasive diagnostics or procedures causing psychological, spiritual or social harm or discomfort for the participants were not involved.

### Sample characteristics

Our study sample consisted of N = 1494 German adults aged 18–89, all of them being involved in a heterosexual relationship and cohabiting with their partner. Relationship duration was between 6 month and 67 years (M = 25.6, SD = 15.8 years). Most participants were married, (n = 1309 - 87.6%), while some were unmarried (n = 129 - 8.6%), few were divorced or living in divorce (n = 43 - 2.9%) or widowed (n = 13 - 0.9%). Gender distribution was equally balanced with n = 759 (50.8%) females. Mean age was 52.0 years (*SD* = 15.26). Based on age, three almost equal sized groups were formed: The younger group (n = 508 - 34%), i.e., 18–44 year old, the middle-aged group (n = 518 – 34.6%), i.e., the 45–60 year old, and the group of the elderly, i.e., 61 year old and older participants (n = 468 - 31.4%). With respect to educational level, n = 655 (43.8%) received lower secondary education, n = 571 (38.2%) secondary education, n = 268 (17.9%) had a high school degree. The majority of participants (n = 1119 - 74.8%) had joint children with their current partner, the mean number of children was M = 1.95 (*SD* = .89).

### Measurements

#### Quality of Relationship Inventory

The QRI is a self-report questionnaire consisting of 25 items that are evaluated on a 4-point Likert scale ranging from *1 = not true* to 4* = almost always true*. According to Pierce et al. [9] the 25 items yield three dimensions: *Support* (7 items, e.g., ‘To what extent could you turn to this person for advice about problems?’), *conflict* (12 items, e.g., ‘How often do you have to work hard to avoid conflict with this person?’), and *depth* (6 items, e.g., ‘How significant is this relationship in your life?’). The QRI takes about 5 minutes to administer. The German version of QRI was translated and back-translated from the English Original by native speakers. In order to ensure clarity and comprehensibility, the final German version of the QRI was pre-tested in a sample of 30 German psychology students.

#### Assessment of importance/significance of differential relationship aspects

Participants indicated the importance as well as their general satisfaction on four items dealing with basic relationship aspects: Importance and satisfaction with family/children and with relationship/sexuality. Importance and satisfaction were evaluated on a 5-point Likert scale ranging from *1 = very unsatisfied* to 5* = very satisfied*, respectively. Although sexuality and relationship satisfaction are somehow distinct, previous research has shown that these two domains are highly interrelated in non-single individuals [28], [29].

## Results

### Research Question 1: Confirming the three factor structure by comparison of three competing models of the QRI

We investigated the factor structure of the QRI by comparing 3 different models by means of confirmatory factor analysis (CFA) according to Verhofstadt and colleagues [24]. The three models had an increasing number of factors and thereby an increasing complexity or higher resolution in measuring relationship quality. We started from a one-factor model, in which the 25 items were assumed to be indicators of a single latent construct, i.e., quality of relationship (Model A). The second model, Model B, was the oblique two factor model found by Nakano et al. [25]. In a sample of 40 Japanese childless couples who had had two recurrent spontaneous abortions, a two-factor structure was found, in which 14 support items and 11 conflict items were assumed to measure two correlated latent constructs (i.e., support and conflict). Model C was our target model and consisted of an oblique three-factor model proposed by Pierce et al. [8]. Thereby, seven items loaded on the support dimension, 12 items on the conflict dimension, and six items on the depth dimension, respectively.

#### Statistical Analyses

To determine how many latent factors were needed to account for variation among the QRI data, we examined three alternative models described above using confirmatory factor analyses (CFA) performed with Mplus 5.1 [30]. The maximum likelihood method of estimation was used to fit the models. Correlations between the latent variables were permitted (analogous to an oblique rotation). Nonzero error covariances between the observed variables were not allowed in the models tested in the current study. Each item of the QRI was allowed to load freely on its hypothesized factor but was not allowed to load on other factors. Models were evaluated on several indices of goodness of fit. The overall fit of the models was evaluated using the χ^2^-test, the comparative fit index (CFI), and the root mean square error of approximation (RMSEA). According to current conventions, goodness of fit is indicated by a non-significant χ^2^- value, a CFI/TLI greater than .95, and an RMSEA below .05 [31]. In a large sample, as in the current study, the chi-square test statistic will nearly always be significant, even when there are good fitting models [32]. Therefore, chi-square divided by degree of freedom is also reported. In general, the ratio of Chi^2^ to df should be smaller than 2 or 3 [31]. The Akaike information criterion is a modification of the standard goodness-of-fit Chi^2^ statistic that accounts for the complexity of the model [33]. When comparing several competing models, the one with the lowest model Akaike information criterion is preferred [34].

As Table 1 shows, the results of the three goodness-of-fit analyses suggest that the three-factor structure of the QRI proposed by Pierce et al. [8] best fits our data. The three-factor solution possesses the lowest Akaike information criterion (AIC = 65,403.03) of all three estimated models. Due to the sample size of 1.494 the Chi^2^ test statistic is significant, although the other fit indices indicate a very good fit to our data: The ratio of Chi^2^ to df with Chi^2^/df = 2.04 is smaller than 3, CFI and TLI are both greater than .95, and both, the RMSEA as well as the SRMR, are below .05. By contrast, the one-factor model as well as the two-factor model inadequately fit our data.

**Table 1:** Goodness-of-Fit-Indices for the Different Models.

	Fit indices							
Model	x^2^	df	X^2^/df	CFI	TLI	AIC	RMSEA	SRMR
Model A	2483.49*	224	11.09	.857	.809	67,428.61	.084	.089
Model B	2073.96*	223	9.30	.883	.843	67,021.07	.076	.084
Model C	451.92*	221	2.04	.985	.980	65,403.03	.027	.029

Note. CFUI = comparative fit index, TLI = Tucker-Lewis index; AIC = Akaike information criterion; RMSEA = root-mean-sqaure error of approximation; SRMR = standardized root-mean-square residual; Model A = one-factor model; Model B = oblique two-factor model of Nakano et al. (2002); Model C = oblique three-factor model of Pierce et al. (1991).

* *p*<.001.

In addition to the overall model fit, the components of fit were examined as well. All standardized factor loadings of the QRI items in the tree-factor solution were significant (see Table 2). Further, results indicate that the three scales of the QRI have a good reliability with all α's above .82.

**Table 2:** Standardized Factor Loading for Each item of the Quality of Relationship Inventory (QRI) in the Oblique Three-Factor Solution.

Item	Support	Conflict	Depth
1. To what extent could you turn to this person for advice about problems?	.73		
2. How often do you have to work hard to avoid conflict with this person?		.64	
3. To what extent could you count on this person for help with a problem?	.76		
4. How upset does this person sometimes make you feel?		.69	
5. To what extent can you count on this person to give you honest feedback, even uif you might not want to hear it?	.55		
6. How much does this person make you feel guilty?		.65	
7. How much do you have to “give in” in this relationship?		.62	
8. To what extent can you count on this person to help you if a family member very close to you died?	.71		
9. How much does this person want you to change?		.74	
10. How positive a role does this person play in your life?			.79
11. How significant is this relationship in your life?			.81
12. How close will your relationship be with this person in 10 years?			.81
13. How much would you miss this person if the two of you could not see or talk with each other for a month?			.75
14. How critical of you is this person?		.44	
15. If you wanted to go out and do something this evening, how confident are you that this person would be willing to do something with you?	.50		
16. How responsible do you feel for this person's well-being?			.48
17. How much do you depend on this person?			.33
18. To what extent can you count on this person to listen to you when you are very angry at someone else?	.65		
19. How much would you like this person to change?		.76	
20. How angry does this person make you feel?		.77	
21. How much do you argue with this person?		.62	
22. To what extent can you really count on this person to distract you from your worries when you feel under stress?	.70		
23. How often does this person make you feel angry?		.71	
24. How often does this person try to control or influence your life?		.67	
25. How much more do you give than you get from this relationship?		.32	
Cronbach's α	.841	.888	.824

Note. All standardized factor loading had significant *t* values (*p*≤.001). QRI items reproduced from Pierce et al.'s (1991) study.

### Research Question 2: External Validation with differential relationship aspects

We investigated the association between the QRI scales and four items exploring importance and satisfaction with respect to the children/family domain and relationship/sexuality domain, respectively. As the QRI assesses the subjectively perceived quality of individual's (romantic) relationship, we expected – compared to items assessing importance of differential relationship domains or satisfaction with children or family issues – the highest correlations between “satisfaction with relationship/sexuality” and the QRI scales.

First, we report descriptive characteristics of the four items assessing different relationship aspects. Further, importance and satisfaction indices with different relationship domains (family/children and relationship/sexuality) were linked with the QRI scales.

#### Different Relationship Aspects – Descriptive characteristics

With respect to the degree of importance, “children and family” was important to all participants (M = 4.33, SD = .83) as well as the “relationship and sexuality” domain (M = 4.06, SD = .92). There was a significant difference between females and males: Women (M = 4.40, SD = .78) reported higher importance of children/family issues than men (M = 4.26, SD = .88; *F*(1, 1458) = 11.201, *p≤*.001). Further, no gender difference with respect to importance of relationship and sexuality aspects could be found. With respect to the degree of satisfaction, the total sample reported a very high satisfaction with respect to children/family issues (M = 4.23, SD = .78) as well as with relationship/sexuality aspects (M = 4.08, SD = .88). No gender differences emerged in the reported satisfaction in these two basic domains. Age was negatively associated with both relationship/sexuality importance (r_pm_ = −.29, *p≤*.001) and satisfaction (r_pm_ = −.15, *p≤*.001). The correlation between age and importance and significance of the children/family domain was non-significant.

#### Validation of the QRI and Different Relationship Aspects

The scale scores for the three QRI scales support, conflict and depth were computed by averaging the scores on the corresponding items. Intercorrelations of the QRI scales as well as the correlation between these three scales and the external criteria of importance and satisfaction with different relationship domains are portrayed in Table 3. In line with theoretical assumptions and previous empirical findings, support and depth are highly positively correlated, while support and conflict, as well as depth and conflict are highly negatively correlated with each other.

**Table 3:** Intercorrelations of the QRI scales and correlations with of importance and satisfaction with different relationship domains.

	QRI Support	QRI Conflict	QRI Depth
QRI Conflict	−.46		
QRI Depth	.74	−.41	
Importance Children/Family	.26	−.19	.26
Importance Relationship/Sexuality	.32	−.13	.27
Satisfaction Children/Family	.35	−.29	.33
Satisfaction Relationship/Sexuality	.45	−.35	.42

Note. All correlations were significant, *p≤*.001.

Overall, the three QRI scales are significantly correlated with the reported importance and satisfaction with different relationship domains. Support and depth are positively correlated with each indicator of importance and satisfaction, while conflict is negatively associated with importance and satisfaction. As expected, the association between the QRI scales and satisfaction is continuously higher than the correlation with importance.

### Research Question 3: Report of psychometric properties (differences regarding age, sex and parenthood) of the QRI scales (Support, Conflict, and Depth)

Next, we investigated sex and age differences as well as differences between parents and childless participants with respect to the three QRI scales support, conflict, and depth. To date, there is no information on QRI scales' characteristics in a German sample. QRI sample characteristics from other countries mostly stem from rather small or student or clinical samples. We hereby provide descriptive data on the QRI scales in a large representative sample of German individuals who have been in a cohabiting heterosexual relationship.

#### Descriptive Characteristics of the QRI scales

As portrayed in Table 4, participants described their relationship with their partner as deep, highly supportive, and low in conflict in our total sample. With respect to sex differences and controlled for age, females perceived and described their relationships as less supportive than males (*F*(1, 1493) = 14.21, *p≤*.001). No significant sex differences emerged regarding the conflict and depth scales. Age was significantly correlated with conflict (r_pm_ = −.08, *p≤*.005) but not with support and depth. With respect to the three age groups, elderly participants perceived their relationships as significantly less conflicted (*F*(1, 1493) = 5.44, *p≤*.004)., than the younger (posthoc: *p≤*.001), and the middle-aged groups (posthoc: *p≤*.010), while differences between the younger and the middle-aged group do not reach significance. Relationship duration is - controlled for age - significantly positively related to perceived support (r_par_ = .08, *p≤*.005) and depth (r_par_ = .15, *p≤*.001), and negatively associated with conflict (r_par_ = −.14, *p≤*.001). Further, having common children or not is also (controlled for age and sex) significantly associated with the overall quality of relationship, that is perceived support (*F*(1, 1493) = 5.04, *p = *  = 025), the conflict (*F*(1, 1493) = 19.02, *p≤*.001) and depth (*F*(1, 1493) = 23.50, *p≤*.001): Participants who had common children with their partner report higher perceived support and depth of the relationship as well as less conflict than individuals with no common children or childless participants. No significant interactions between age groups, sex and parenthood with respect to the three QRI scales were found.

**Table 4:** QRI Scales: Means and Standard Deviations (Total sample, Sex and Age groups, Parenthood).

		Support		Conflict		Depth	
		M	*SD*	M	*SD*	M	*SD*
**Total sample**	*n* = 35	3.20	0.61	1.83	0.49	3.27	0.54
**Sex**							
*Females*	*n* = 759	3.15	0.58	1.84	0.49	3.25	0.54
*Males*	*n* = 735	3.26	0.53	1.82	0.48	3.30	0.54
**Age Groups**							
*Age 18–44*	*n* = 508	3.23	0.57	1.87	0.52	3.25	0.55
*Age 45–60*	*n = *518	3.17	0.55	1.84	0.49	3.26	0.52
*Age 61–89*	*n = *468	3.22	0.56	1.76	0.44	3.33	0.54
**Parenthood**							
*Children*	*n* = 1136	3.22	0.54	1.79	0.46	3.31	0.54
*No Children*		3.15	0.60	1.94	0.56	3.15	0.53

## Discussion

The major aim of study 1 was to investigate the factor structure of the QRI by means of confirmatory factor analysis (CFA) in a sample of German adults. In line with previous research, the findings of study 1 support the proposed three-factor structure of Pierce et al [8]. Our approach was very similar to the one of Verhofstadt and colleagues [24] by comparing three competing models of the QRI. We were able to replicate and expand their findings to a sample of German non-couples with a wide age range (from age 18 to 89). Replication of the findings of Verhofstadt and colleagues [24] does not only include confirming a three-factor structure of the QRI but also refers to comparable internal consistencies, comparable intercorrelations between the scales, and even further, comparable standardized factor loadings. For example, Verhofstadt and colleagues [24] reported internal consistencies ranging from α = .80 to α = .88 for males and α = .79 to α = .87 for females, respectively. Internal consistencies calculated on the basis of our sample range from α = .82 to α = .89. Moreover, the standardized factor loadings of the QRI items on the three scales are about the same. The reported intercorrelations of the three dimensions were almost equal our findings [24]. Similar intercorrelation patterns have been reported from US samples [10], supporting the assumption that perceptions of support in a particular relationship are indeed different from, but related to, perceptions of conflict and depth in that relationship [8]. In turn, our findings contradict the two factor solution proposed by Nakano et al. [25] These differences in factor structure might be due to cultural differences or, more likely, to the specific sample of childless couples who have undergone particular personal and relationship distress (two recurrent spontaneous abortions) in the Japanese sample. In our representative sample, however, findings concerning the substantial interrelatedness of support, conflict, and depth perceptions further support the claim made by Pierce et al [8] that partners' perceptions of several features of their romantic relationship form a coherent view of the quality of this specific relationship.

In line with previous results on relationship satisfaction and QRI scales, our findings propose high criterion validity. Correlations between the three QRI scales and relationships-concerned “satisfaction items” were higher than correlations between relationships-concerned “importance items”. The association between the QRI scales and the item “satisfaction of relationship/sexuality” was the strongest and higher than correlations with other relationships-concerned items, indicating both high discriminate and construct validity.

Finally, our study provides precious information about demographic data on relationship quality in a representative sample of German women and men being in a cohabiting relationship. Overall, the relationship quality in cohabiting persons is fairly high. Independent of age, however, women perceive less support in the relationship by their partner. Previous studies on gender differences in perceived support produced mixed results; one study with American undergraduate students suggested that females perceive the relationship less conflicted and more supportive than males [10]. In contrast, another study with an elderly sample (age 57 to 85) underlines our findings suggesting that women feel less supported in the relationship than men [35]. Likely, these contradictory findings might be due to relationship duration or living/home status. Further research needs to explore gender differences in relationship quality in cohabiting couples versus couples living apart. Regarding age influences on relationship quality, our study further supported previous findings that conflict in relationship decreases with age [36] which is possibly due to a selection process that older participants maintained longer in a relationship. Interestingly, both effects of sex and age are main effects, and no interactions between sex and age emerged with respect to the three QRI scales. Moreover and in line with intuition, relationship duration is positively associated with the relationship quality: The longer the relationship lasts, the more supportive the partner is perceived and the deeper and less conflicted the relationship is described. Our study further revealed that participants who had at least one child with their current partner perceived their relationship as more supportive, deeper and less conflicted than participants with no joint children in their current relationship. This surprising finding remains stable also after controlled for age, age groups and sex and contradicts previous studies that propose common children as a “relationship stressor”, especially in early stages of parenthood [37], [38]. It should be noted that we cannot state whether our sample participants of non-parents were generally childless or had no joint children with their current partner, but children from previous unions. Thus, in our sample of “non-parent” cohabiting individuals, participants living in patchwork arrangements and those with no children are confounded. Further research needs to explore whether the lower overall perceived relationship quality is due to childlessness or possible difficulties due to patchwork arrangements.

In conclusion, our findings provide new empirical evidence for the factorial validity of the QRI. On the basis of the present and previous findings [8], [24], we recommend a further use of the three-factor structure of the QRI (i.e., support, conflict, and depth). We first reported psychometric and descriptive characteristics on the basis of a large representative German sample. Our findings both complement as well as elaborate on existing theory and research on relationship quality and social support.

### Limitations

There are two significant limitations to this study that suggest future research. First, a potential limitation of the current study deals with the choice of the sample in which we investigated the factor structure and its invariance. All of our participants were cohabiting and involved in a heterosexual romantic relationship which was obviously predominantly well-functioning. Further, the majority of our participants were married. It remains unclear whether our findings can be generalized to participants who are involved in a relationship but living apart or who live in a more conflicted relationship (e.g., seeking marital counseling) or who are involved in a same-sex relationship. Therefore, it is important for future studies to determine whether the pattern of results found in the present study can be replicated in more diverse samples with respect to sexual orientation and variations in relationship satisfaction. A second limitation is the cross-sectional design of the study. It is not possible to differentiate between selection or causation processes with respect to (a) importance or satisfaction with different relationship domains, (b) sex and age, (c) the fact of having children. It should be noted that this was not a goal of the present study.
